# Immune checkpoint inhibitor exposure and outcomes of gastrointestinal bleeding in cancer patients: a national analysis of 130,557 hospitalizations, 2018–2022

**DOI:** 10.3389/fmed.2026.1869656

**Published:** 2026-07-17

**Authors:** Wael Alkattan, Mohammed Barnawi, Omar Chalabi, Maamon Mallisho, Tarek Ziad Arabi, Marwan Alaswad, Abderrahman Ouban

**Affiliations:** College of Medicine, Alfaisal University, Riyadh, Saudi Arabia

**Keywords:** cancer, gastrointestinal bleeding, immune checkpoint inhibitors, immune-related adverse events, in-hospital mortality, National Inpatient Sample

## Abstract

**Background:**

Although immune checkpoint inhibitors (ICIs) have transformed cancer treatment, they are associated with immune-related adverse events, including gastrointestinal (GI) toxicity. While GI bleeding is a common cause of hospitalization among cancer patients, the relationship between ICI exposure and the outcomes of GI bleeding at the national level remains unclear.

**Methods:**

This retrospective cohort study utilized data from the National Inpatient Sample (NIS) between 2018 and 2022. Adult cancer patients hospitalized with GI bleeding were identified and stratified by ICI exposure. The primary outcome was in-hospital mortality. Secondary outcomes included colectomy, intensive care unit (ICU) admission, and blood transfusion. Survey-weighted logistic regression models, adjusted for demographics, insurance, income, admission type, and year, were employed. Sensitivity analyses included adjustments for Elixhauser comorbidities, inverse probability of treatment weighting (IPTW), propensity score matching, exclusion of records with inpatient antineoplastic chemotherapy codes, exclusion of records from pandemic years, and alternative ICU-proxy thresholds.

**Results:**

Of the 130,557 hospitalizations, 33,130 (25.4%) involved ICI exposure. ICI exposure was associated with significantly lower in-hospital mortality (adjusted odds ratio [aOR] 0.57, 95% confidence interval [CI] 0.54–0.60, *p* < 0.001), colectomy (aOR 0.65, 95% CI 0.60–0.70, *p* < 0.001), and ICU admission (aOR 0.66, 95% CI 0.64–0.68, *p* < 0.001). Blood transfusion was modestly more frequent in the ICI group (aOR 1.04, 95% CI 1.00–1.08, *p* = 0.034); this association was small in magnitude and was no longer significant when the pandemic years were excluded. The results were consistent across cancer subtypes, GI bleeding locations, and all sensitivity analyses (mortality aOR range 0.57–0.66).

**Conclusion:**

ICI exposure was associated with significantly lower in-hospital mortality, colectomy, and ICU admission among cancer patients hospitalized with GI bleeding. These findings likely reflect selection bias, as ICI-treated patients may represent a healthier subpopulation of cancer patients. Further studies incorporating detailed clinical data are needed to elucidate the mechanisms underlying these associations.

## Introduction

Immune checkpoint inhibitors (ICIs) have fundamentally transformed the landscape of cancer therapeutics over the past decade. By blocking inhibitory receptors on T cells—principally cytotoxic T-lymphocyte-associated protein 4 (CTLA-4), programmed cell death protein 1 (PD-1)—and programmed death-ligand 1 (PD-L1), ICIs restore antitumor immune surveillance and have demonstrated durable responses across a broad spectrum of malignancies (1, 2). Since the approval of ipilimumab for metastatic melanoma in 2011, the number of ICI indications has rapidly expanded, and it is currently estimated that more than 40% of patients with cancer in the United States are eligible for ICI-based therapy (3, 4).

However, by augmenting immune activation, ICIs carry a distinct risk of immune-related adverse events (irAEs) that can affect virtually any organ system. Meta-analyses have reported an overall irAE incidence of approximately 27% for anti-PD-1/PD-L1 monotherapy and up to 60% for combination regimens, with gastrointestinal (GI) toxicity ranking among the most common and clinically consequential manifestations (5, 6). ICI-related GI toxicity encompasses a spectrum ranging from mild diarrhea to severe colitis, enterocolitis, and, in rare cases, life-threatening perforation or hemorrhage (7, 8). Current guidelines from the American Society of Clinical Oncology (ASCO) and the Society for Immunotherapy of Cancer (SITC) recommend prompt recognition and graded management of GI irAEs, including corticosteroids and biologic agents such as infliximab for refractory cases (9, 10).

Gastrointestinal bleeding is a common and serious cause of hospitalization among patients with cancer. The etiology of GI bleeding in this population is multifactorial, encompassing tumor-related hemorrhage, treatment-related mucosal injury, coagulopathy, and preexisting GI pathology (11, 12). Cancer patients who present with GI bleeding experience higher mortality and longer hospitalizations than non-cancer patients, reflecting the compounding effects of advanced disease, immunosuppression, and comorbid conditions (13). Nationally, GI bleeding accounts for hundreds of thousands of U. S. hospitalizations annually, with significant associated morbidity, mortality, and healthcare costs (14, 15).

Despite the expanding use of ICIs and the known risk of GI toxicity, the relationship between ICI exposure and clinical outcomes among cancer patients hospitalized with GI bleeding remains unexamined at the national level. It remains unknown whether ICI-exposed patients experience different rates of mortality, colectomy, ICU admission, or blood transfusion compared to non-ICI cancer patients with GI bleeding. Understanding this relationship is clinically relevant, as it may inform risk stratification, resource allocation, and management decisions for this growing patient population.

Therefore, we conducted a nationally representative retrospective cohort study using the National Inpatient Sample (NIS) between 2018 and 2022 to evaluate the association between ICI exposure and in-hospital outcomes among cancer patients hospitalized with GI bleeding. We hypothesized that ICI exposure would be associated with different clinical outcomes compared with those of non-ICI cancer patients, and we further examined whether these associations varied by cancer type and GI bleeding location.

## Methods

### Study design and data sources

This was a retrospective, cross-sectional study using data from the National Inpatient Sample (NIS) from 2018 to 2022. The NIS is the largest publicly available all-payer inpatient database in the United States, maintained by the Healthcare Cost and Utilization Project (HCUP) and sponsored by the Agency for Healthcare Research and Quality (AHRQ) (16, 17). It represents a 20% stratified sample of all discharges from community hospitals nationwide, with each record assigned a discharge weight (DISCWT) that enables the generation of national estimates. The NIS contains clinical and resource-use information abstracted from hospital discharge records, including diagnoses coded using the International Classification of Diseases, Tenth Revision, Clinical Modification (ICD-10-CM), and procedures coded using the ICD-10 Procedure Coding System (ICD-10-PCS). Because the NIS contains only publicly available de-identified data, this study was exempt from institutional review board approval requirements.

### Study population

The study population comprised adult patients (age 18 years or older) hospitalized with GI bleeding and a concurrent diagnosis of malignancy between January 2018 and December 2022. GI bleeding was identified using ICD-10-CM codes for hematemesis (K92.0), melena (K92.1), unspecified GI hemorrhage (K92.2), gastric and duodenal ulcers with hemorrhage (K25.0, K25.2, K25.4, K25.6, K26.0, K26.2, K26.4, K26.6), peptic and gastrojejunal ulcers with hemorrhage (K27.0–K28.6), gastritis with bleeding (K29.01), esophageal varices with bleeding (I85.01, I85.11), hemorrhage of the anus and rectum (K62.5), angiodysplasia of the colon with hemorrhage (K55.21), and diverticular disease with hemorrhage (K57.x1, K57.x3). Cancer was defined as the presence of any ICD-10-CM code ranging from C00 to C96 in any diagnostic field. Patients with missing data regarding in-hospital mortality, discharge weight, length of stay, or total charges were excluded.

### Exposure definition

The primary exposure was ICI therapy, identified using a combination of ICD-10-CM codes Z79.899 (long-term use of other medications), Z51.11 (encounter for antineoplastic chemotherapy), and Z92.21 (personal history of antineoplastic chemotherapy). This combination served as a proxy for ICI exposure in the context of an active cancer diagnosis because ICI-specific billing codes were not available in the ICD-10-CM during the study period (2018–2022). Patients with cancer with GI bleeding who met these criteria constituted the ICI group; all remaining patients with cancer with GI bleeding formed the non-ICI comparator group. Because these Z-codes are not specific to ICIs and can also be assigned to patients receiving conventional cytotoxic chemotherapy, we performed a sensitivity analysis excluding hospitalizations with markers of inpatient antineoplastic chemotherapy administration (ICD-10-PCS 3E0 antineoplastic introduction codes and new-technology antineoplastic codes) or chemotherapy-induced agranulocytosis (D70.1). The likely direction of any residual exposure misclassification is addressed in the “Discussion” section. The limited specificity of administrative codes for identifying specific drug exposures is well recognized (18).

### Outcome measures

The primary outcome was in-hospital mortality. Secondary outcomes included colectomy (identified using ICD-10-PCS codes 0DTE, 0DTN, 0DTP, 0DTF, 0DTG, and 0DTH), intensive care unit (ICU) admission (because the NIS does not contain a direct indicator of ICU or critical care unit use during the data years; ICU-level care was approximated using a composite proxy defined as a length of stay of 3 days or longer, together with total hospital charges of $50,000 or more, with sensitivity analyses using alternative thresholds assessed as described below), and blood transfusion (identified using ICD-10-PCS section 302 transfusion codes encompassing whole blood, packed and frozen red blood cells, platelets, and plasma products across all vascular access routes and both autologous and non-autologous qualifiers; the original three-code definition [30,233 N1, 30,243 N1, 30,233 L1] was retained for comparison). Additional secondary outcomes included length of stay and total hospital charges.

### Covariates

The covariates included age (continuous), sex (female vs. male patients), primary payer (Medicare, Medicaid, private insurance, self-pay, no charge, or other), median household income for the patient’s ZIP code by national quartile, admission type (emergency vs. non-emergency), and discharge year (2018–2022). The cancer subtypes were classified as lung, melanoma, renal, bladder, colorectal, or other based on ICD-10-CM diagnosis codes. GI bleeding location was categorized as upper, lower, or unspecified/other based on specific ICD-10-CM codes present. The Elixhauser comorbidity index was calculated from ICD-10-CM diagnosis codes using van Walraven weights (19–21), yielding a single continuous summary score. Individual Elixhauser flags for coagulopathy, liver disease, alcohol abuse, renal failure, congestive heart failure, and metastatic cancer were also extracted for use in propensity score models. Ethnicity was excluded as a covariate due to the known incompleteness of NIS ethnicity data across participating states during the study period.

### Statistical analysis

Baseline characteristics were compared between the ICI and non-ICI groups using survey-weighted descriptive statistics. Continuous variables were reported as weighted mean (standard deviation), or median [interquartile range], and categorical variables were reported as weighted frequencies (percentages). Between-group comparisons were performed using two-sample *t*-tests for means, Wilcoxon rank-sum tests for medians, and chi-square tests for proportions. Given the large number of baseline comparisons, the *p*-values in Table 1 were additionally adjusted for multiple comparisons using the Benjamini-Hochberg false discovery rate method.

**Table 1:** Baseline characteristics of cancer patients with GI bleeding by ICI exposure.

Variable	ICI + cancer (*N* = 33,130)	Non-ICI cancer (*N* = 97,427)	*p*-value
Age (years), mean (SD)	68.4 (13.3)	69.9 (13.0)	< 0.001
Female patients, *n* (%)	14,112 (42.6%)	40,090 (41.2%)	< 0.001
Length-of-stay (days), median [IQR]	5 [3–8]	6 [3–11]	< 0.001
Total charges ($), median [IQR]	51,494 [29,022–98,503]	65,729 [33,922–134,876]	< 0.001
Mortality, *n* (%)	2,588 (7.8%)	12,518 (12.8%)	< 0.001
Colectomy, *n* (%)	924 (2.8%)	3,989 (4.1%)	< 0.001
ICU admission, *n* (%)	15,844 (47.8%)	55,887 (57.4%)	< 0.001
Blood transfusion, *n* (%)	11,562 (34.9%)	33,309 (34.2%)	0.019
Emergency admission, *n* (%)	30,615 (92.4%)	90,806 (93.2%)	< 0.001

ICI, immune checkpoint inhibitor; SD, standard deviation; IQR, interquartile range; ICU, intensive care unit.

Ethnicity data are not included due to the known incompleteness of NIS ethnicity data across participating states (2018–2022).

The association between ICI exposure and each binary outcome was evaluated using survey-weighted logistic regression models (generalized linear models with a binomial family and logit link function). Discharge weights (DISCWTs) were applied as variance weights to account for the NIS sampling design without inflating the effective sample size. Cluster-robust standard errors, clustered by the hospital identifier (HOSP_NIS), were calculated to account for within-hospital correlation arising from the stratified cluster sampling design. All models were adjusted for age, sex, primary payer, income quartile, admission type, and discharge year. The results are reported as adjusted odds ratios (aORs) with 95% confidence intervals (CIs).

To examine whether the association between ICI exposure and in-hospital mortality differed by cancer subtype, separate survey-weighted logistic regression models were fitted within each cancer subgroup (lung, melanoma, renal, bladder, colorectal, and other). For subgroups in which any cell in the two-by-two outcome-by-exposure table contained fewer than 10 events, Firth’s penalized logistic regression was applied to mitigate sparse-data bias and quasi-complete separation bias (22). Separate weighted logistic regression models for mortality were fitted across upper, lower, and unspecified/other GI bleeding subgroups, with a formal interaction test (ICI exposure × GI bleeding location) conducted by including a product term in the model.

### Sensitivity analyses

Several prespecified sensitivity analyses were conducted. First, the primary regression models were re-estimated by adding the Elixhauser comorbidity score as a continuous covariate, alongside the original adjustment variables. Second, inverse probability of treatment weighting (IPTW) was performed using a propensity score for ICI exposure estimated using logistic regression analysis, with age, sex, payer, income quartile, admission type, year, the Elixhauser comorbidity score, individual Elixhauser flags, and GI bleeding location considered covariates. Stabilized weights were trimmed at the first and 99th percentiles and multiplied by the NIS discharge weights to generate combined weights (23). Third, 1:1 nearest-neighbor propensity score matching without replacement was performed using a caliper of 0.2 standard deviations of the logit of the propensity score (24). The propensity score model included 29 covariates in total. Covariate balance was assessed using standardized mean differences (SMDs), with adequate balance defined as all absolute SMDs being < 0.10 across all covariates. Additional sensitivity analyses included (i) excluding hospitalizations with inpatient antineoplastic chemotherapy markers, (ii) excluding the peak pandemic years (2020–2021 and, separately, 2020 alone), (iii) formally testing a calendar year by ICI interaction on mortality, and (iv) re-estimating the ICU outcome under alternative length of stay and charge thresholds. Finally, the melanoma subgroup, limited by sparse events, was analyzed using Firth’s penalized regression and is reported in Supplementary Table S1.

All statistical analyses were performed using the statsmodels and SciPy libraries in Python 3. A two-sided *p*-value of <0.05 was considered statistically significant.

## Results

### Study population

**Figure 1:**
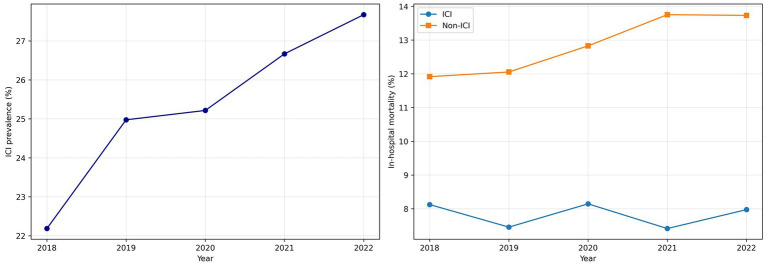
Temporal trends in immune checkpoint inhibitor (ICI) exposure and in-hospital mortality among cancer patients hospitalized with gastrointestinal bleeding, National Inpatient Sample, 2018–2022. (Left) Annual prevalence of ICI exposure, rising from 22.2% in 2018 to 27.7% in 2022. (Right) Annual in-hospital mortality by ICI exposure status; mortality remained stable (7.4–8.1%) in the ICI group and was consistently higher in the non-ICI group.

Between 2018 and 2022, 130,557 hospitalizations for GI bleeding in cancer patients were identified in the NIS, of which 33,130 (25.4%) involved documented ICI exposure. The proportion of GI bleeding hospitalizations among cancer patients involving ICI exposure increased from 22.2% in 2018 to 27.7% in 2022 (Figure 1).

### Baseline characteristics

Baseline characteristics of the study cohort are presented in Table 1. Patients in the ICI group were slightly younger than those in the non-ICI group (mean age 68.4 vs. 69.9 years, *p* < 0.001) and a higher proportion of female patients (42.6% vs. 41.2%, *p* < 0.001). The ICI group also had a shorter median length of stay (5 vs. 6 days, *p* < 0.001) and incurred lower median total hospital charges ($51,494 vs. $65,729, *p* < 0.001). Emergency admissions accounted for the majority of hospitalizations in both groups (92.4% vs. 93.2%, *p* < 0.001).

### Primary outcomes

**Figure 2:**
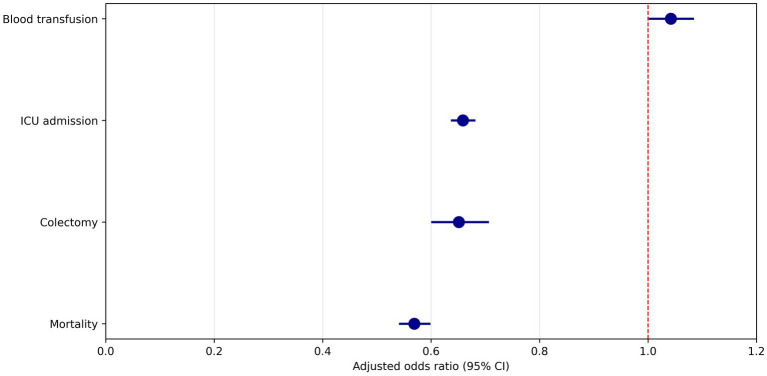
Forest plot of adjusted odds ratios (95% CI) for in-hospital mortality, colectomy, ICU admission, and blood transfusion associated with ICI exposure, from survey-weighted multivariable logistic regression. The vertical dashed line marks an odds ratio of 1.

In the primary survey-weighted logistic regression analysis, after adjusting for age, sex, insurance type, household income quartile, admission type, and year of admission, ICI exposure was associated with significantly lower odds of in-hospital mortality (adjusted odds ratio [aOR] 0.57, 95% confidence interval [CI] 0.54–0.60, *p* < 0.001), colectomy (aOR 0.65, 95% CI 0.60–0.70, *p* < 0.001), and ICU admission (aOR 0.66, 95% CI 0.64–0.68, *p* < 0.001) (Table 2; Figure 2). Blood transfusions were modestly more frequent in the ICI group, reaching nominal statistical significance (aOR 1.04, 95% CI 1.00–1.08, *p* = 0.034); however, the magnitude of this association was small and of uncertain clinical importance. Using the original three-code transfusion definition, the corresponding estimate was an aOR of 1.05 (95% CI 1.02–1.10, *p* = 0.006), and expanding the procedure codes to include whole blood and additional red cell products (as outlined in the Methods section) did not materially alter this finding.

**Table 2:** Multivariable-adjusted outcomes for ICI vs. non-ICI cancer patients with GI bleeding.

Outcome	Adjusted OR	95% CI	*p*-value
In-hospital mortality	0.57	0.54–0.60	< 0.001
Colectomy	0.65	0.60–0.70	< 0.001
ICU admission	0.66	0.64–0.68	< 0.001
Blood transfusion	1.04	1.00–1.08	0.034

Adjusted for age, sex, payer, income quartile, admission type, year, Elixhauser comorbidities, cancer type, and GI bleed location.

### Temporal trends

The prevalence of ICI exposure in this cohort increased steadily over the study period, rising from 22.2% in 2018 to 27.7% in 2022 (Figure 1, left panel). In-hospital mortality in the ICI group remained relatively stable, ranging from 7.4 to 8.1% across all years. In contrast, mortality in the non-ICI group showed an upward trend, increasing from approximately 11.9% in 2018 to 13.7% in 2022 (Figure 1, right panel). A formal test for interaction between calendar year and ICI exposure was statistically significant (*p* = 0.015), indicating that the relative mortality difference between groups widened over the study period; consequently, year-stratified estimates are presented in Figure 1.

### Subgroup analysis by cancer type

**Figure 3:**
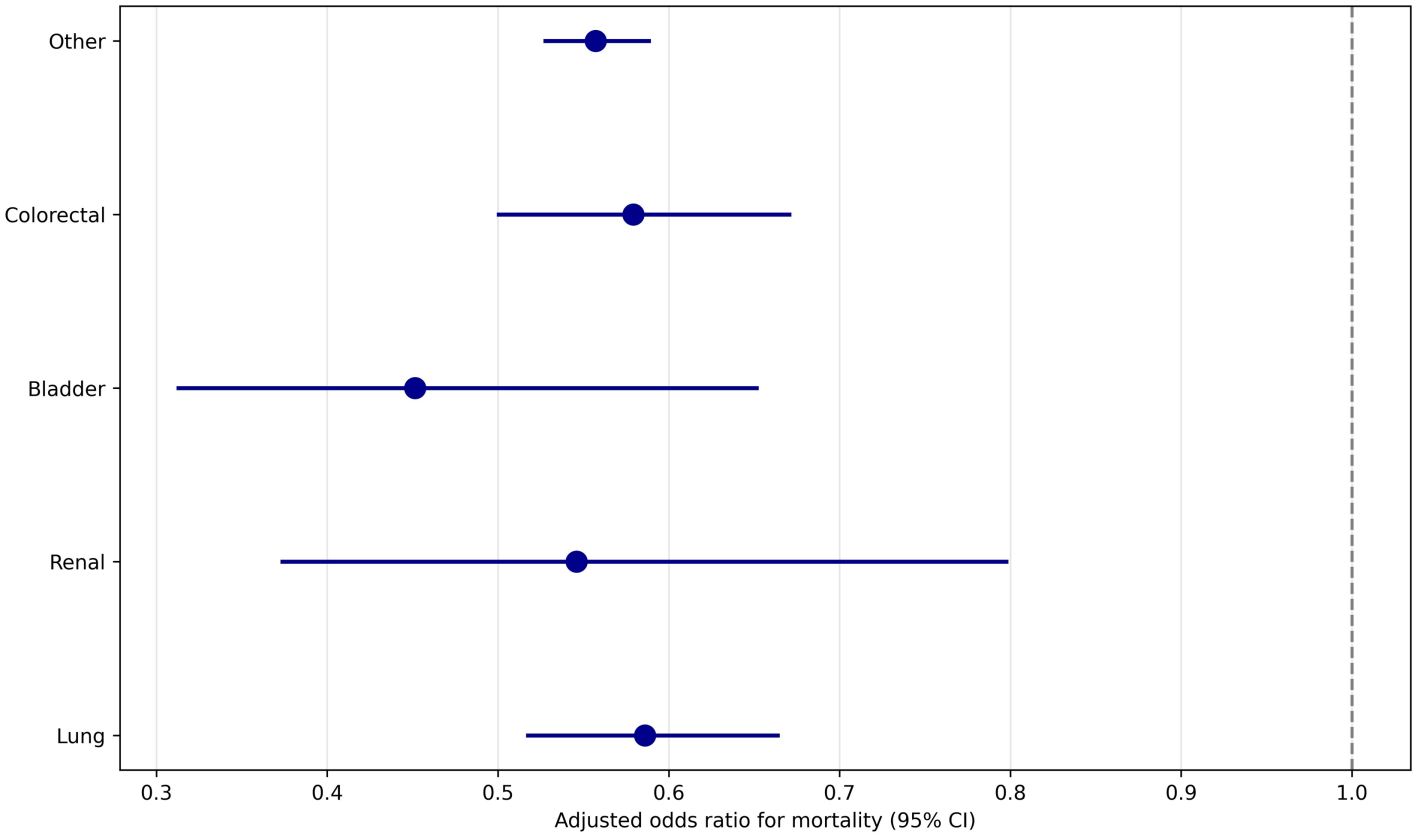
Forest plot of adjusted odds ratios (95% CI) for in-hospital mortality associated with ICI exposure, stratified by cancer type (lung, renal, bladder, colorectal, other). The vertical dashed line marks an odds ratio of 1.

The association between ICI exposure and in-hospital mortality was examined across cancer subtypes (Table 3; Figure 3). Statistically significant reductions in mortality were observed among patients with lung cancer (aOR 0.59, 95% CI 0.52–0.66, *p* < 0.001), renal cell carcinoma (aOR 0.55, 95% CI 0.37–0.80, *p* = 0.002), bladder cancer (aOR 0.45, 95% CI 0.31–0.65, *p* < 0.001), colorectal cancer (aOR 0.58, 95% CI 0.50–0.67, *p* < 0.001), and other cancer types (aOR 0.56, 95% CI 0.53–0.59, *p* < 0.001). The melanoma subgroup was limited by sparse events and was therefore analyzed using Firth’s penalized logistic regression; given its instability, it is reported separately in Supplementary Table S1 and should be interpreted with caution.

**Table 3:** Mortality by cancer type subgroup.

Cancer type	*N*	*N* (ICI)	Adjusted OR	95% CI	*p*-value	Method
Lung	12,262	3,086	0.59	0.52–0.66	< 0.001	Weighted GLM
Renal	2,985	588	0.55	0.37–0.80	0.002	Weighted GLM
Bladder	2,789	618	0.45	0.31–0.65	< 0.001	Weighted GLM
Colorectal	21,840	5,092	0.58	0.50–0.67	< 0.001	Weighted GLM
Other	87,264	22,928	0.56	0.53–0.59	< 0.001	Weighted GLM

The melanoma subgroup is reported in Supplementary Table S1 (sparse events; Firth penalized regression). Cancer subgroups are mutually exclusive; the melanoma subgroup is shown in Supplementary Table S1.

### Subgroup analysis by GI bleeding location

Analysis by GI bleeding location revealed consistent associations between ICI exposure and lower in-hospital mortality for both upper GI bleeding (aOR 0.58, 95% CI 0.55–0.61, *p* < 0.001) and lower GI bleeding (aOR 0.52, 95% CI 0.44–0.60, *p* < 0.001) (Table 4). The subgroup with unspecified or other GI bleeding locations demonstrated a similar association (aOR 0.52, 95% CI 0.34–0.79, *p* = 0.002). The test for interaction between GI bleeding location and ICI exposure was not statistically significant (*p* for interaction = 0.135), indicating that the association between ICI exposure and mortality was consistent across bleeding sites.

**Table 4:** Mortality by GI bleed location subgroup.

GI location	*N*	*N* (ICI)	Adjusted OR	95% CI	*P*-value
Upper	103,000	25,791	0.58	0.55–0.61	< 0.001
Lower	22,521	6,129	0.52	0.44–0.60	< 0.001
Unspecified/Other	2,620	636	0.52	0.34–0.79	0.002
Interaction p-value					0.135

24 patients (4 ICI) with missing GI bleed location were excluded from the subgroup totals.

### Sensitivity analyses

Three prespecified sensitivity analyses were performed to assess the robustness of the primary findings.

First, in a model adjusting for the Elixhauser Comorbidity Index in addition to the primary covariates, the results remained consistent for mortality (aOR 0.65, 95% CI 0.62–0.68, *p* < 0.001), colectomy (aOR 0.62, 95% CI 0.57–0.67, *p* < 0.001), and ICU admission (aOR 0.70, 95% CI 0.68–0.72, *p* < 0.001). Blood transfusion was also nominally significant in this model (aOR 1.06, 95% CI 1.02–1.10, *p* = 0.003) (Table 5).

**Table 5:** Elixhauser comorbidity score-adjusted outcomes.

Outcome	Adjusted OR	95% CI	*p*-value
Mortality	0.65	0.62–0.68	< 0.001
Colectomy	0.62	0.57–0.67	< 0.001
ICU admission	0.70	0.68–0.72	< 0.001
Transfusion	1.06	1.02–1.10	0.003

Adjusted for the Elixhauser comorbidity score (continuous) rather than for individual comorbidity indicators.

Second, the IPTW analysis yielded similar results: mortality (aOR 0.64, 95% CI 0.61–0.67, *p* < 0.001), colectomy (aOR 0.72, 95% CI 0.66–0.78, *p* < 0.001), ICU admission (aOR 0.72, 95% CI 0.70–0.74, *p* < 0.001), and blood transfusion (aOR 1.06, 95% CI 1.02–1.10, *p* = 0.002) (Table 6).

**Table 6:** Inverse probability of treatment weighting (IPTW) outcomes.

Outcome	IPTW OR	95% CI	*p*-value
Mortality	0.64	0.61–0.67	< 0.001
Colectomy	0.72	0.66–0.78	< 0.001
ICU admission	0.72	0.70–0.74	< 0.001
Transfusion	1.06	1.02–1.10	0.002

Propensity score weights stabilized and trimmed at the first and 99th percentiles.

**Figure 4:**
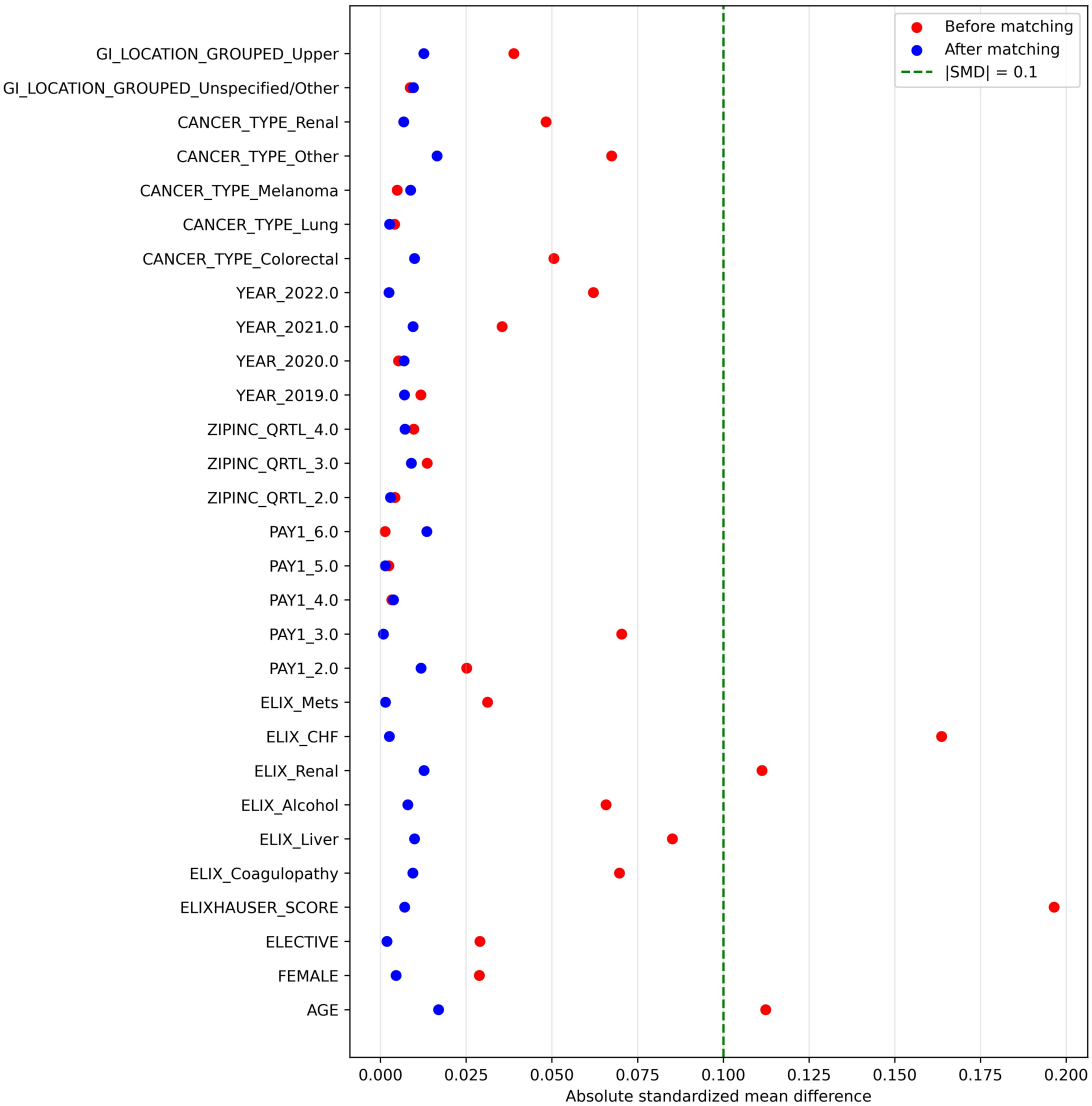
Love plot of absolute standardized mean differences for baseline covariates before (red) and after (blue) 1:1 propensity score matching; the vertical dashed line at 0.10 denotes the balance threshold.

Third, 1:1 propensity score matching without replacement yielded 32,556 matched pairs. After matching, all 29 assessed covariates, including age, sex, the Elixhauser Comorbidity Index, individual comorbidities (e.g., coagulopathy, liver disease, alcohol use disorder, renal disease, congestive heart failure, and metastatic disease), insurance type, household income quartile, year of admission, cancer type, and GI bleeding location, achieved adequate balance with all absolute standardized mean differences below 0.10 (Table 7; Figure 4). In the matched cohort, ICI exposure remained associated with significantly lower in-hospital mortality (7.8% vs. 11.3%, OR 0.66, 95% CI 0.63–0.70, *p* < 0.001), lower colectomy rates (2.8% vs. 3.7%, OR 0.74, 95% CI 0.67–0.80, *p* < 0.001), and lower ICU admission rates (47.8% vs. 55.9%, OR 0.72, 95% CI 0.70–0.74, *p* < 0.001) (Table 8). Blood transfusion rates were marginally higher in the matched analysis (34.9% vs. 34.0%, OR 1.04, 95% CI 1.01–1.08, *p* = 0.011).

**Table 7:** Propensity score-matched cohort: baseline characteristics and covariate balance.

Variable	ICI (*N* = 32,556)	Non-ICI (*N* = 32,556)	SMD (Before)	SMD (After)
Age (years), mean	68.5	68.7	−0.112	−0.017
Female patients, %	42.7	42.5	0.029	0.005
Elective admission, %	7.5	7.4	0.029	0.002
Elixhauser score, mean	19.0	18.9	−0.197	0.007
Coagulopathy, %	20.8	20.4	−0.070	0.009
Liver disease, %	15.4	15.1	−0.085	0.010
Alcohol abuse, %	5.6	5.8	−0.066	−0.008
Renal failure, %	21.5	20.9	−0.111	0.013
Congestive heart failure, %	17.6	17.5	−0.164	0.003
Metastatic cancer, %	40.3	40.2	0.031	0.001
Year 2022, %	22.5	22.6	0.062	−0.002
Colorectal cancer, %	15.6	15.3	−0.051	0.010
Lung cancer, %	9.5	9.4	−0.004	0.003
Upper GI bleed, %	79.2	79.7	−0.039	−0.013

SMD, standardized mean difference; ICI, immune checkpoint inhibitor.

All post-matching SMDs < 0.10, indicating adequate balance.

**Table 8:** Propensity score-matched cohort: clinical outcomes.

Outcome	ICI (%)	Non-ICI (%)	OR	95% CI	*p*-value
Mortality	7.8	11.3	0.66	0.63–0.70	< 0.001
Colectomy	2.8	3.7	0.74	0.67–0.80	< 0.001
ICU admission	47.8	55.9	0.72	0.70–0.74	< 0.001
Transfusion	34.9	34.0	1.04	1.01–1.08	0.011

Matched 1:1 on propensity score using nearest-neighbor matching with a caliper of 0.2 SD of the logit.

Across all analytic approaches, in-hospital mortality odds ratios ranged from 0.57 to 0.66, colectomy odds ratios from 0.62 to 0.74, and ICU admission odds ratios from 0.66 to 0.72, respectively, with all estimates remaining statistically significant. Blood transfusion showed a small but consistently positive association across methods (aOR 1.04–1.06), reaching nominal statistical significance in the primary, Elixhauser-adjusted, IPTW, and propensity score-matched analyses; however, the magnitude of the association was small, and the association attenuated to non-significance when the pandemic years of 2020–2021 were excluded (aOR 1.02, *p* = 0.45).

## Discussion

In this nationally representative study of 130,557 cancer patients hospitalized with GI bleeding from 2018 through 2022, we found that ICI exposure was associated with significantly lower odds of in-hospital mortality (aOR 0.57), colectomy (aOR 0.65), and ICU admission (aOR 0.66). These associations remained robust across multiple analytic approaches, including Elixhauser-adjusted models, IPTW, and propensity score matching, with the mortality odds ratios ranging narrowly from 0.57 to 0.66. The proportion of these hospitalizations involving ICI exposure increased from 22.2 to 27.7% over the study period, reflecting the expanding use of ICI therapy in oncology (3, 4).

The finding that ICI-exposed patients experienced lower mortality and fewer adverse outcomes is counterintuitive, given that ICIs are known to cause GI toxicity, including colitis and, in rare cases, hemorrhage (7, 8, 25). ICI-induced colitis occurs in approximately 1–2% of patients receiving anti-PD-1/PD-L1 monotherapy and up to 7% of those receiving ipilimumab-based regimens, with severe cases requiring hospitalization, infliximab, or colectomy (8, 26). Recent real-world analyses have reported higher rates of clinically significant gastrointestinal toxicity than earlier trial-based estimates; for example, a large real-world cohort reported a colitis incidence of approximately 7 per 100 person-years among patients receiving ICIs for non-small-cell lung cancer (27). However, ICI-related colitis is a distinct clinical entity from other causes of GI bleeding in cancer patients, characterized by specific histopathological features and a generally favorable response to immunosuppressive therapy (28, 29).

Importantly, the NIS cannot distinguish gastrointestinal bleeding caused by immune-related colitis from the many other causes of bleeding in cancer patients, including tumor-related hemorrhage, anticoagulation, thrombocytopenia, portal hypertension, radiation injury, and peptic ulcer disease. Therefore, our findings describe associations between ICI exposure and the outcomes of gastrointestinal bleeding of any cause and should not be interpreted as reflecting the natural history or management of ICI-induced colitis specifically.

The most plausible explanation for our findings is healthy user bias—also referred to as channeling bias or confounding by indication—whereby ICI-treated patients represent a systematically healthier subpopulation of cancer patients (30). To be eligible for and tolerate ICI therapy, patients must typically have an adequate performance status, preserved organ function, and a disease trajectory compatible with ongoing systemic therapy. In contrast, the non-ICI group in our study likely includes a disproportionate number of patients with advanced, treatment-refractory, or end-stage disease who are no longer candidates for active cancer-directed therapy. The NIS does not capture performance status, disease stage, line of therapy, specific ICI agent, the timing of therapy relative to the bleeding event, goals of care, or use of immunosuppressive therapy, and these unmeasured confounders are likely responsible for a substantial portion of the observed association. Although our sensitivity analyses—including Elixhauser comorbidity adjustment, IPTW, and propensity score matching—attenuated the effect marginally (mortality aOR increasing from 0.57 to 0.66), the persistent association across all approaches suggests residual confounding that administrative data cannot fully address.

The temporal trends observed in our study provide additional context. Mortality in the ICI group remained relatively stable at 7.5–8.0% over the study period, whereas mortality in the non-ICI group trended upward from approximately 11.9 to 13.7% between 2018 and 2022. Furthermore, the formal calendar-year by ICI exposure interaction was statistically significant (*p* = 0.015). This divergence may reflect an evolving composition of the non-ICI group. As the progressive expansion of ICI indications shifts an increasing proportion of fitter cancer patients into the ICI-exposed category, the non-ICI group becomes enriched for patients with a poorer baseline prognosis. Wang et al. have reported high mortality among hospitalized patients receiving inpatient ICI therapy; although that study did not specifically examine gastrointestinal bleeding, it underscores the fact that the hospitalized ICI-treated population is heterogeneous and can be of high acuity (31). These observations are consistent with the expanding body of real-world literature characterizing the incidence, risk factors, and outcomes of immune-related adverse events in routine practice (32, 33).

The consistent association across cancer subtypes strengthens the internal validity of our findings. Statistically significant reductions in mortality were observed for lung cancer, renal cell carcinoma, colorectal cancer, and other cancer types. The melanoma subgroup showed a large but unstable effect estimate (aOR 0.27; Supplementary Table S1), which should be interpreted with caution given the small event count and reliance on Firth penalized regression. Similarly, the association was consistent across upper and lower GI bleeding locations, with a non-significant interaction test (*p* = 0.135), suggesting that the observed effect was not driven by a specific bleeding etiology.

Blood transfusion was marginally more frequent in the ICI group across analyses (aOR 1.04–1.06), reaching statistical significance in the primary model (*p* = 0.034) and sensitivity analyses. However, the absolute difference was small (approximately one percentage point in the matched cohort); this association attenuated to non-significance when the pandemic years 2020–2021 were excluded (aOR 1.02, *p* = 0.45), and the NIS does not capture transfusion thresholds or volumes. We therefore interpret this small positive association cautiously and do not consider it indicative of a clinically meaningful difference in bleeding severity between groups.

### Limitations

This study has several limitations inherent to the use of administrative data. First, the NIS does not contain ICI-specific billing codes, and our proxy-based exposure definition, which relies on Z-codes for antineoplastic therapy, may misclassify some patients, likely resulting in non-differential misclassification that biases the results toward the null. The accuracy of ICD-10 codes for identifying specific medication exposures in administrative databases remains a known limitation (34). Second, the NIS lacks key clinical variables, including cancer stage, performance status, line of therapy, specific ICI agent, and severity of GI bleeding, all of which are important confounders that could not be measured or adjusted for (16, 17). Third, the NIS does not contain a direct ICU admission indicator during the study period, and ICU-level care was approximated using a composite proxy based on length of stay and total charges; this proxy is partly circular with illness severity and resource use, although results were similar across alternative thresholds. Fourth, because the NIS is an admission-level rather than patient-level database, patients with multiple hospitalizations may be counted more than once, which is particularly relevant for cancer patients and may affect estimates of exposure prevalence and precision; furthermore, associations observed at the hospitalization level may not translate directly to individual patients (i.e., the ecological fallacy). Fifth, the NIS captures inpatient data only and does not allow for the assessment of post-discharge outcomes, readmissions, or longitudinal follow-up. Finally, despite extensive sensitivity analyses, residual confounding—particularly from unmeasured performance status and disease severity—cannot be excluded and likely accounts for a substantial portion of the observed associations.

## Conclusion

In this nationally representative study, ICI exposure was associated with significantly lower rates of in-hospital mortality, colectomy, and ICU admission among cancer patients hospitalized with GI bleeding. These findings are most consistent with healthy user bias, whereby ICI-treated patients represent a systematically healthier subgroup of cancer patients. Consequently, these results should not be interpreted as evidence that ICI therapy is protective against adverse outcomes in GI bleeding. Rather, they highlight the importance of accounting for treatment selection effects in observational studies of ICI outcomes and underscore the need for prospective studies incorporating detailed clinical data, including cancer stage, performance status, specific ICI agent, timing of therapy relative to bleeding, and validated measures of GI bleeding severity, to elucidate the true relationship between ICI therapy and GI bleeding outcomes in cancer patients.

## Data Availability

The datasets presented in this study can be found in online repositories. The names of the repository/repositories and accession number(s) can be found at agency for Healthcare Research and Quality (AHRQ) Healthcare Cost and Utilization Project (HCUP) National Inpatient Sample (NIS). Access requires a data use agreement; further information is available at: https://www.hcup-us.ahrq.gov.
