# Supplementary material for: Immune checkpoint inhibitor exposure and outcomes of gastrointestinal bleeding in cancer patients: a national analysis of 130,557 hospitalizations, 2018–2022
Source: Front Med (Lausanne). 2026 Jul 17;13:1869656. doi: 10.3389/fmed.2026.1869656 (PMC13423715; doi:10.3389/fmed.2026.1869656)
Supplement: Supplementary file 1 [file Table_1.DOCX]

**Supplementary Table S1. Mortality in the melanoma subgroup (sparse events; Firth penalized logistic regression).**

| Cancer Type | N | N (ICI) | Adjusted OR | 95% CI | P-value | Method |
| --- | --- | --- | --- | --- | --- | --- |
| Melanoma | 1,022 | 246 | 0.27 | 0.13–0.58 | <0.001 | Firth |
