# Supplementary material for: Immune checkpoint inhibitor exposure and outcomes of gastrointestinal bleeding in cancer patients: a national analysis of 130,557 hospitalizations, 2018–2022
Source: Front Med (Lausanne). 2026 Jul 17;13:1869656. doi: 10.3389/fmed.2026.1869656 (PMC13423715; doi:10.3389/fmed.2026.1869656)
Supplement: Supplementary file 2 [file Table_2.DOCX]

**Table S2.** Propensity Score Covariate Balance

| **Covariate** | **SMD Before Matching** | **SMD After Matching** |
| --- | --- | --- |
| Age (continuous) | -0.1123 | -0.0170 |
| Female sex | 0.0288 | 0.0045 |
| Elective admission | 0.0290 | 0.0019 |
| Elixhauser Comorbidity Score (continuous) | -0.1965 | 0.0070 |
| Coagulopathy (Elixhauser) | -0.0697 | 0.0094 |
| Liver Disease (Elixhauser) | -0.0852 | 0.0099 |
| Alcohol Use Disorder (Elixhauser) | -0.0658 | -0.0080 |
| Renal Failure (Elixhauser) | -0.1113 | 0.0127 |
| Congestive Heart Failure (Elixhauser) | -0.1637 | 0.0026 |
| Metastatic Cancer (Elixhauser) | 0.0312 | 0.0014 |
| Payer: Medicaid | 0.0252 | 0.0118 |
| Payer: Private Insurance | 0.0704 | 0.0008 |
| Payer: Self-Pay | -0.0033 | -0.0038 |
| Payer: No Charge | 0.0024 | -0.0014 |
| Payer: Other | -0.0014 | 0.0135 |
| Median Household Income Quartile 2 | 0.0042 | 0.0029 |
| Median Household Income Quartile 3 | -0.0136 | -0.0090 |
| Median Household Income Quartile 4 | -0.0097 | -0.0072 |
| Discharge Year: 2019 | -0.0118 | -0.0070 |
| Discharge Year: 2020 | -0.0053 | 0.0069 |
| Discharge Year: 2021 | 0.0355 | 0.0095 |
| Discharge Year: 2022 | 0.0621 | -0.0025 |
| Cancer Type: Colorectal | -0.0506 | 0.0099 |
| Cancer Type: Lung | -0.0041 | 0.0026 |
| Cancer Type: Melanoma | -0.0049 | 0.0088 |
| Cancer Type: Other | 0.0674 | -0.0165 |
| Cancer Type: Renal | -0.0483 | 0.0068 |
| GI Bleeding Location: Unspecified/Other | -0.0087 | -0.0096 |
| GI Bleeding Location: Upper | -0.0389 | -0.0126 |

*SMD, standardized mean difference. Values below 0.1 indicate adequate covariate balance after propensity score matching.*
